# Combination of Dexmedetomidine and Tramadol in Patient-Controlled Intravenous Analgesia Strengthens Sedative Effect in Pregnancy-Induced Hypertension

**DOI:** 10.3389/fphar.2021.739749

**Published:** 2021-10-22

**Authors:** Shu-Yao Zhang, Hui Zhao, Chengcheng Xu, Qiuzhen Zhang, Yun Chen, Hai-Yan Li, Xia-Lan Zhang, Chengkuan Zhao, Meini Chen, Dong-Hua Yang

**Affiliations:** ^1^ Department of Pharmacy, Guangzhou Red Cross Hospital Jinan University, Guangzhou, China; ^2^ Department of Gynecology and Obstetrics, The Second Affiliated Hospital of Shantou University Medical College, Shantou, China; ^3^ Department of Pharmacology, Shantou University Medical College, Shantou, China; ^4^ Department of Nursing, Guangzhou Red Cross Hospital Jinan University, Guangzhou, China; ^5^ Department of Pharmaceutical Sciences, St. John’s University College of Pharmacy and Health Sciences, Queens, NY, United States

**Keywords:** dexmedetomidine, inflammation, interleukin 10, P38-mitogen-activated protein kinase, pregnancy-induced hypertension, sedative effect, tramadol

## Abstract

**Objective:** The aim of the present study is to explore the combination of dexmedetomidine (DXM) and tramadol (TMD) on sedative effect in patients with pregnancy-induced hypertension (PIH).

**Methods:** A total of 356 patients with pregnancy-induced hypertension (PIH) were randomly divided into three groups: DXM, TMD and DXM + TMD groups. These patients were treated with different doses of DXM, TMD or combination of DXM and TMD by a patient-controlled intravenous injection device. The scores of static pain and dynamic pain, sedation degree, and adverse reaction were recorded. The plasma levels of inflammatory mediators IL-10 and C-reactive protein (CRP), and the serum level of p-p38-MAPK were evaluated.

**Results:** It was found that administration with DXM 1.0 µg/kg/h + TMD 700 mg and DXM 2.0 µg/kg/h + TMD 600 mg result in stronger sedative effect than single administration with DXM or TMD. The mean arterial pressure (MAP) and heart rate (HR) of patients with PIH were decreased with the combinational treatment of DXM and TMD. Interestingly, the PIH patients injected with DXM 1.0 µg/kg/h + TMD 700 mg and DXM 2.0 µg/kg/h + TMD 600 mg showed stronger sedative effect. In addition, the plasma level of level of IL-10 was increased and CRP decreased. The serum level of p-p38/MAPK was decreased.

**Conclusion:** Taken together, our study indicates that combination of DXM and TMD effectively lowers blood pressure and reduces inflammation through increasing the level of IL-10, reducing CRP and inhibiting p-p38/MAPK in patients with PIH. This study suggests that the combination of DXM and TMD could be an anesthetic choice in the management of PIH.

## Introduction

Pregnancy-induced hypertension (PIH) refers to a state where diastolic blood pressure is higher than 90 mmHg, or in the situation where systolic blood pressure is higher than 140 mmHg when tested in not less than 2 times with the duration of not less than 4 h each time (Veerbeek et al., 2015). PIH is a major factor leading to maternal, embryonic and newborn morbidity and mortality (Kintiraki et al., 2015). Currently, there is no effective management for PIH except early delivery of the baby (Han et al., 2018a). Therefore, it is a pressing need to explore methods for management of PIH.

Hypertensive diseases happen in pregnant state with an incidence of 10% worldwide. It is important to identify the risk factors for PIH and relieve the burden of the illness. Dexmedetomidine (DXM) has been reported to have a positive role in helping control blood pressure as well as uterine bleeding in the process of cesarean section in patients with PIH (Li et al., 2016a; Hariharan, 2017; Kong et al., 2018). Tramadol (TMD) is a synthetic 4-phenyl-piperidine analogue of codeine and possesses a centrally analgesic function (Memarian et al., 2018). TMD is widely used as an analgesic drug for its effectiveness and safety (Perdreau et al., 2015). DXM can reduce renin release, and therefore lowering arterial blood pressure (Soliman and Saad, 2018). DXM could also inhibit blood pressure and heart rate of patients during tracheal intubation (Luthra et al., 2017). DXM administration effectively maintains the hemodynamic stability in patients receiving cesarean section who undergo general anesthesia and does not cause adverse effects to the newborns (Yu et al., 2015). TMD injection suppressed systolic arterial blood pressure in rabbits (Egger et al., 2009). Besides, administration of TMD contributes to efficient analgesia after surgery in cesarean section patients with general anesthesia (Demirel et al., 2014).

The development of PIH is closely associated with inflammation. Interleukin 10 (IL-10) is a critical immunoregulatory factor with important function in suppressing inflammation in immune response (Soliman et al., 2018). IL-10 works in a feedback mechanism for several immune responses including adjusting inflammatory cytokine production and inflammatory cell migration (Harmon et al., 2015). It has been reported that PIH animals showed a decreased IL-10 (Kemse et al., 2016). The P38-mitogen-activated protein kinase (MAPK) functions in various signaling pathways (Wang et al., 2016), and plays a critical role in neurodegenerative diseases as well as inflammatory response as a pain regulator (Lin et al., 2014). It has been determined in rats that p38 MAPK signaling pathway functions in the prevention of intra-abdominal hypertension (Chang et al., 2016).

Patient-controlled intravenous analgesia (PCIA) is an efficient approach for improving analgesic effect. PCIA is characterized by high patient satisfaction, minimal sedation or complications (Ding et al., 2016). In this study, we explore whether combination of DXM and TMD using PCIA could strengthen the sedative effect and alleviate PIH. Because PIH is frequently associated with inflammation, we determine whether combination of DXM and TMD could suppress inflammation by inhibiting IL-10 and other inflammatory factors.

## Materials and Methods

### Study Subjects

A total of 356 patients with PIH who underwent cesarean section in the Department of Gynecology and Obstetrics of the Second Affiliated Hospital of Shantou University Medical College (Shantou, China) from January 2016 to December 2018 were included in the study. Patients aged 23–38 years old, body weight was 66–92 kg. Among these patients, 187 patients were with gestational hypertension, 138 patients with preeclampsia, and 31 patients were in other conditions, such as chronic hypertension, “white-coat” hypertension and others. All the selected patients were diagnosed as PIH without other pregnancy complication, kidney disease or diabetes. The patients had no contraindications for combined spinal-epidural anesthesia and no sinus bradycardia or conduction block confirmed by electrocardiogram. Before anesthesia, the patients and their family members were introduced to the use of PCIA device, the precautions, the Prince-Henry scoring method, the procedure of blood collection and the time of preoperative and postoperative visits.

### Sample Size Estimation

The primary endpoint was comparison of pain score and sedation score of the patients 24 h after surgery. The sample size estimation was conducted based on Fisher hypothesis with 0.05 bilateral alpha and 90% certainty. The sample size calculation also took into account the nine different subgroups in this study.

### Patient Grouping and Drug Intervention

Patients were anaesthetized by the combined spinal-epidural anesthesia with injection of 10 mg 0.5% preservative free ropivacaine in cavum subarachnoid. The epidural local anesthetic was added depending on the length of the operation time. The PCIA device was turned on 15 min before the end of surgery. The PCIA pump has a capacity of 100 ml, a loading dose of 5 ml, a background infusion of 2 ml/h, a Bolous of 0.5 ml and a locking time of 15 min. All 356 patients were randomly allocated into 9 groups according to the dosages and different analgesic methods as 3 DXM sub-groups, 3TMD sub-groups, and 3DXM + TMD sub-groups.

Patients in the DXM group were subdivided into 3 sub-groups (40 cases each) according to the doses as DXM group 1, injected with 0.5 µg/kg/h DXM; DXM group 2, 1.0 µg/kg/h DXM, and DXM group 3, 2.0 µg/kg/h DXM.

Patients in the TMD group were also subdivided into 3 sub-groups (40 cases each) according to the doses as TMD group 1, injected with 600 mg TMD; TMD group 2, 700 mg TMD, and TMD group 3, 800 mg TMD.

Patients in the DXM + TMD group were also subdivided into 3 sub-groups (38–39 cases each) according to the ratio of DXM and TMD. Patients in the DXM + TMD group 1 were injected with 0.5 µg/kg/h DXM + 800 mg TMD, in the DXM + TMD group 2 with 1.0 µg/kg/h DXM + 700 mg TMD, and in the DXM + TMD group 3 with 2.0 µg/kg/h DXM + 600 mg TMD.

### Clinical Evaluation

Adverse events of the subjects in each group were recorded. Adverse events refer to any adverse medical events occurring after signing the informed consent till the last follow-up. Adverse reactions of the patients 24 h after surgery, including nausea, vomiting, drowsiness, respiratory depression et al. were recorded.

The general information of patients in each group including age, body weight, body mass index (BMI) and gestational weeks, was recorded. The Dash3000 multi-parameter monitor was used for recording mean arterial pressure (MAP) and heart rate (HR), respiratory rate (RR) and peripheral oxygen saturation (SpO_2_) before anesthesia, post-surgery 6, 12, 24, and 48 h. Besides, the static pain and dynamic pain scores, sedation score, PCIA total pressing times and invalid pressing times were recorded at 24 h-post surgery.

Prince-Henry scoring method was used for pain evaluation. Prince-Henry scoring is a superior method in objective evaluation of pain because it combines the coughing, breathing and sedative states of patients. Briefly, level 0: no pain when coughing; level 1: pain when coughing; level 2: no pain when quiet, and pain when taking a deep breath; level 3: light pain in a quiet state and could be tolerated; level 4: severe pain in quiet state, which is unbearable (Walaszczyk et al., 2011).

Ramsay scoring method was used for sedation scoring. Briefly, 1 score: not quiet, irritability; 2 scores: quiet and cooperating; 3 scores: lethargy and could follow instructions; 4 scores: sleeping state, but could be woken up; 5 scores: slow respiratory response; 6 scores: deep sleep state, could not be woken up. Among them, 2 to 4 scores indicated satisfactory sedative effect, and 5 to 6 scores represented excessive sedative effect.

### Biological Assays and Outcomes

The venous blood was collected from all patients before anesthesia, 24 and 48 h-post surgery, with 10 ml blood collected each time. One part of the whole blood was collected with an ethylene diamine tetraacetic acid (EDTA) collection tube. The blood sample was naturally coagulated at room temperature for about 20 min, centrifuged at 716 × g for 10 min, and then the supernatant was collected and carefully stored at −70°C. IL-10 in plasma was determined by an ELISA kit (EHC009.48, Shenzhen Neobioscience Technology Co., Ltd, Guangdong, China), and the c-reactive protein content in plasma was detected by the nephelometry (CRP, DE740001,Shenzhen Neobioscience Technology Co., Ltd, Guangdong, China). The other part of the blood was immediately injected into an aseptic tube, centrifuged at 2319 × g for 15 min, and the serum was stored at −20°C. The level of serum p38 MAPK was measured by the human p-p38 MAPK ELISA kit (MBS025490). The above procedures were performed in accordance with the manufacturer’s instructions.

### Statistical Analysis

All data were processed by SPSS 21.0 (IBM Corp. Armonk, NY, United States) statistical software. The normality of numerical data was tested by the Kolmogorov-Smimov test. The Levene method was used to test the homogeneity of variance. The data were presented by mean ± standard deviation. Data with homogeneity of variance among multiple groups were compared by one-way analysis of variance (ANOVA). The comparison of data at different time points among groups were tested by repeated measure ANOVA, and then compared by the Bonferroni method. The measurement data with heterogeneity of variance were analyzed by non-parametric test Kruskal-Wallis H method. The Mann-Whitney U method was used for comparison within groups. Pearson correlation analysis was applied to analyze the correlation among plasma IL-10, CRP and serum p-p38/MAPK in each patient at 24 h-post surgery. The enumeration data were expressed by ratio or percentage, and the chi-square test was used for comparative analysis. *p* < 0.05 indicated statistical difference.

## Results

### General Characteristics of Patients in Different Groups

**FIGURE 1:**
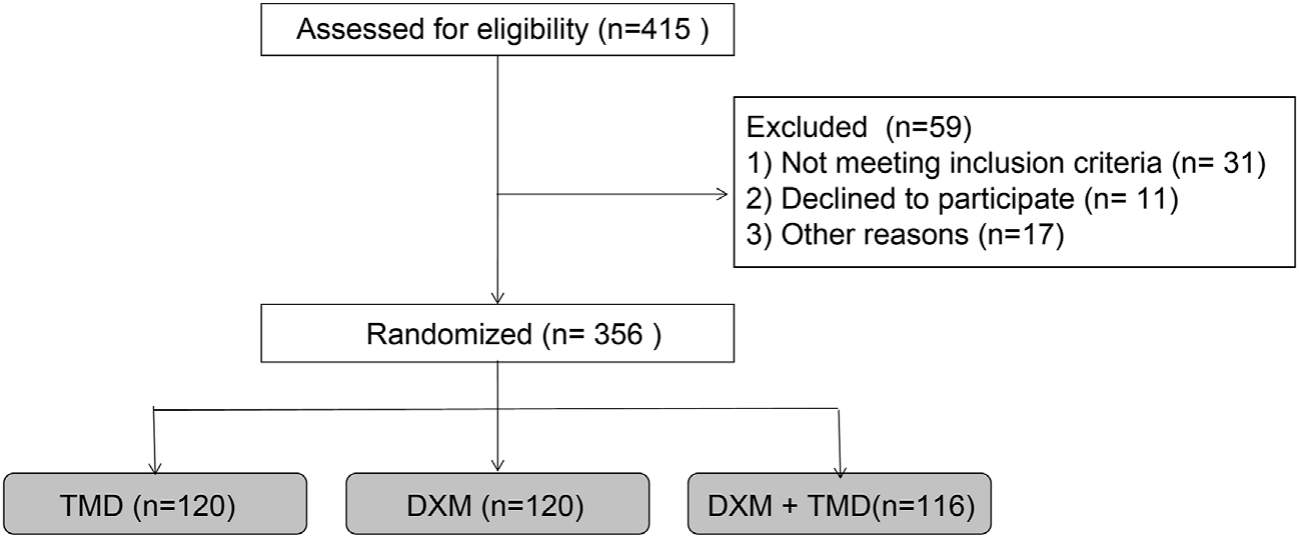
The study flow chart.

The study procedures are shown in Figure 1. The patients with PIH in the DXM group were 29.04 ± 2.94 years old (ranged from 23 to 38 years) and body weight was 79.49 ± 6.16 kg with the BMI of 29.63 ± 2.68 kg/m^2^ and the average gestational week of 38.87 ± 1.52 weeks. The patients with PIH in the TMD group were 29.54 ± 2.23 years old (ranged from 25 to 37 years), had a body weight of 80.16 ± 5.89 kg, a BMI of 30.18 ± 2.69 kg/m^2^, and an average gestational age of 39.06 ± 1.40 weeks. The age of patients with PIH in the DXM + TMD group was 29.33 ± 2.39 years (ranged from 24 to 37 years), the body weight was 79.60 ± 6.11 kg, the BMI was 30.12 ± 2.50 kg/m^2^, and the average gestational age was 39.32 ± 1.41 weeks. There was no difference in age, body weight, BMI and average gestational weeks of patients among these three groups (all *p* > 0.05) (Table 1). Similarly, there was no difference in age, body weight, BMI, and average gestational weeks among the subgroups of DXM group, TMD group, and DXM + TMD group (all *p* > 0.05) (Table 2). These data showed that there was no difference among age, body weight, BMI and gestational age in all patients in different groups.

**TABLE 1:** The general characteristics of patients.

Group	DXM group	TMD group	DXM + TMD group	*p* value
Age (year)	29.04 ± 2.94	29.54 ± 2.23	29.33 ± 2.39	0.311
Body Weight (kg)	79.49 ± 6.16	80.16 ± 5.89	79.60 ± 6.11	0.656
BMI (kg/m^2^)	29.63 ± 2.68	30.18 ± 2.69	30.11 ± 2.50	0.213
Gestational weeks	38.87 ± 1.52	39.06 ± 1.40	39.27 ± 1.40	0.105

Note: DXM group: *n* = 120; TMD group: *n* = 120; DXM + TMD group: *n* = 116; the measurement data were expressed as mean ± standard deviation. Data of multiple groups were compared using one-way ANOVA. BMI, body mass index; DXM, dexmedetomidine; TMD, tramadol.

**TABLE 2:** The general characteristics of patients in each subgroup.

Group	Age (year)	Body Weight (kg)	BMI (kg/m^2^)	Gestational weeks (week)
DXM group 1	29.65 ± 2.82	80.75 ± 6.04	30.13 ± 2.51	39.13 ± 1.47
DXM group 2	28.80 ± 2.85	79.32 ± 6.35	29.23 ± 2.64	38.63 ± 1.43
DXM group 3	28.68 ± 3.54	78.42 ± 9.33	29.54 ± 2.88	38.85 ± 2.83
*p* value	0.313	0.367	0.316	0.541
TMD group 1	29.80 ± 2.10	80.93 ± 5.65	30.67 ± 2.62	39.40 ± 1.30
TMD group 2	29.30 ± 2.19	79.70 ± 6.04	29.86 ± 2.64	38.93 ± 1.56
TMD group 3	29.53 ± 1.41	79.85 ± 6.11	30.00 ± 3.39	38.95 ± 1.41
*p* value	0.513	0.601	0.414	0.254
DXM + TMD group 1	29.69 ± 2.30	80.76 ± 6.07	30.41 ± 2.37	39.54 ± 1.31
DXM + TMD group 2	29.13 ± 2.33	78.84 ± 6.34	30.00 ± 2.54	39.13 ± 1.33
DXM + TMD group 3	29.16 ± 4.24	79.18 ± 6.72	29.92 ± 2.59	39.13 ± 0.71
*p* value	0.667	0.370	0.638	0.257

Note: All subgroups of the DXM and the TMD groups, *n* = 40; the DXM + TMD group 1 and the DXM + TMD 2, *n* = 39; the DXM + TMD group 3, *n* = 38; BMI, body mass index; the measurement data were expressed as mean ± standard deviation. Data of multiple groups were compared using one-way ANOVA; DXM, dexmedetomidine; TMD, tramadol.

### Dexmedetomidine and Tramadol Decreases The Mean Arterial Pressure and Heart Rate of Pregnancy-Induced Hypertension Patients

**FIGURE 2:**
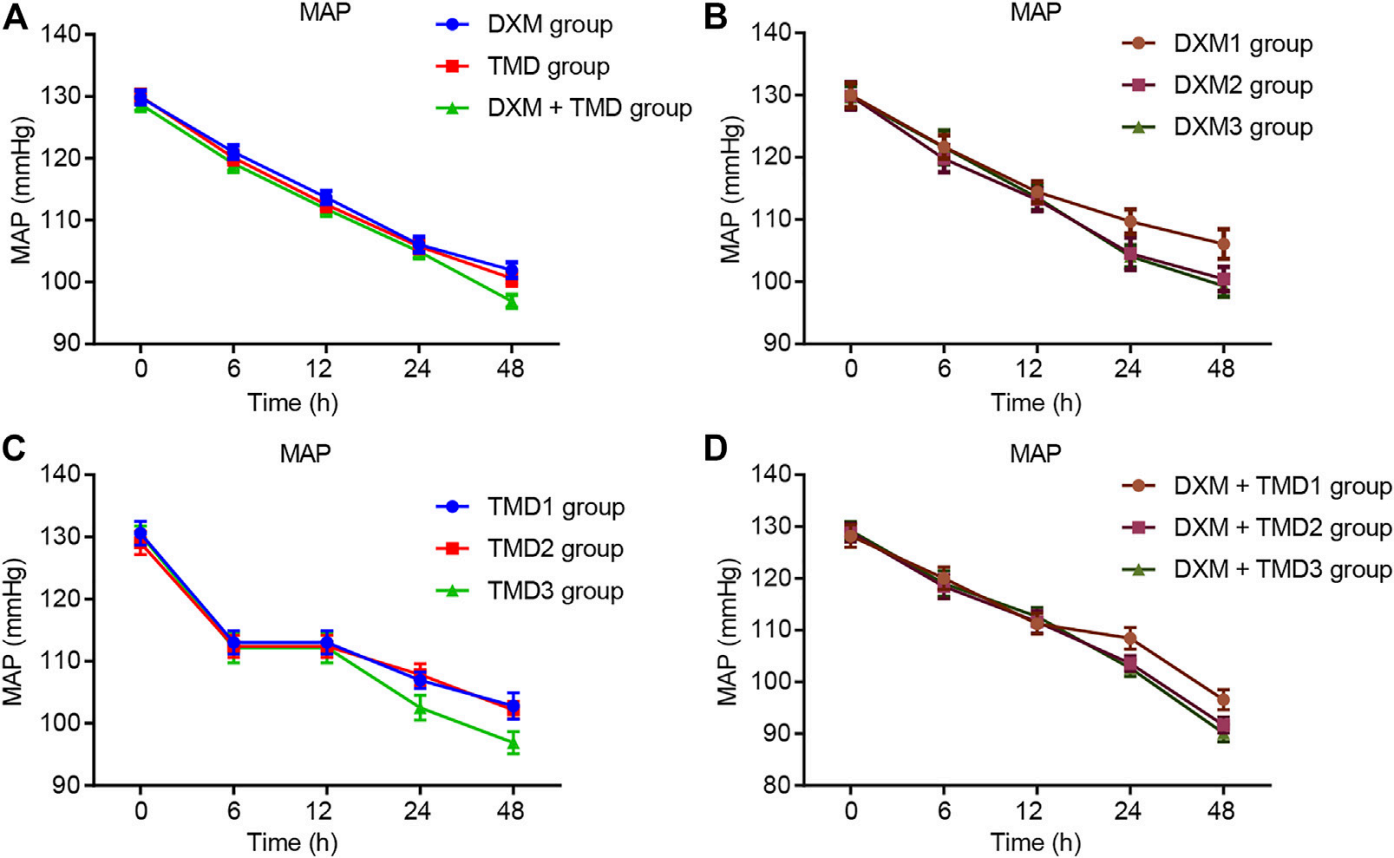
MAP in three groups of patients with PIH.

**FIGURE 3:**
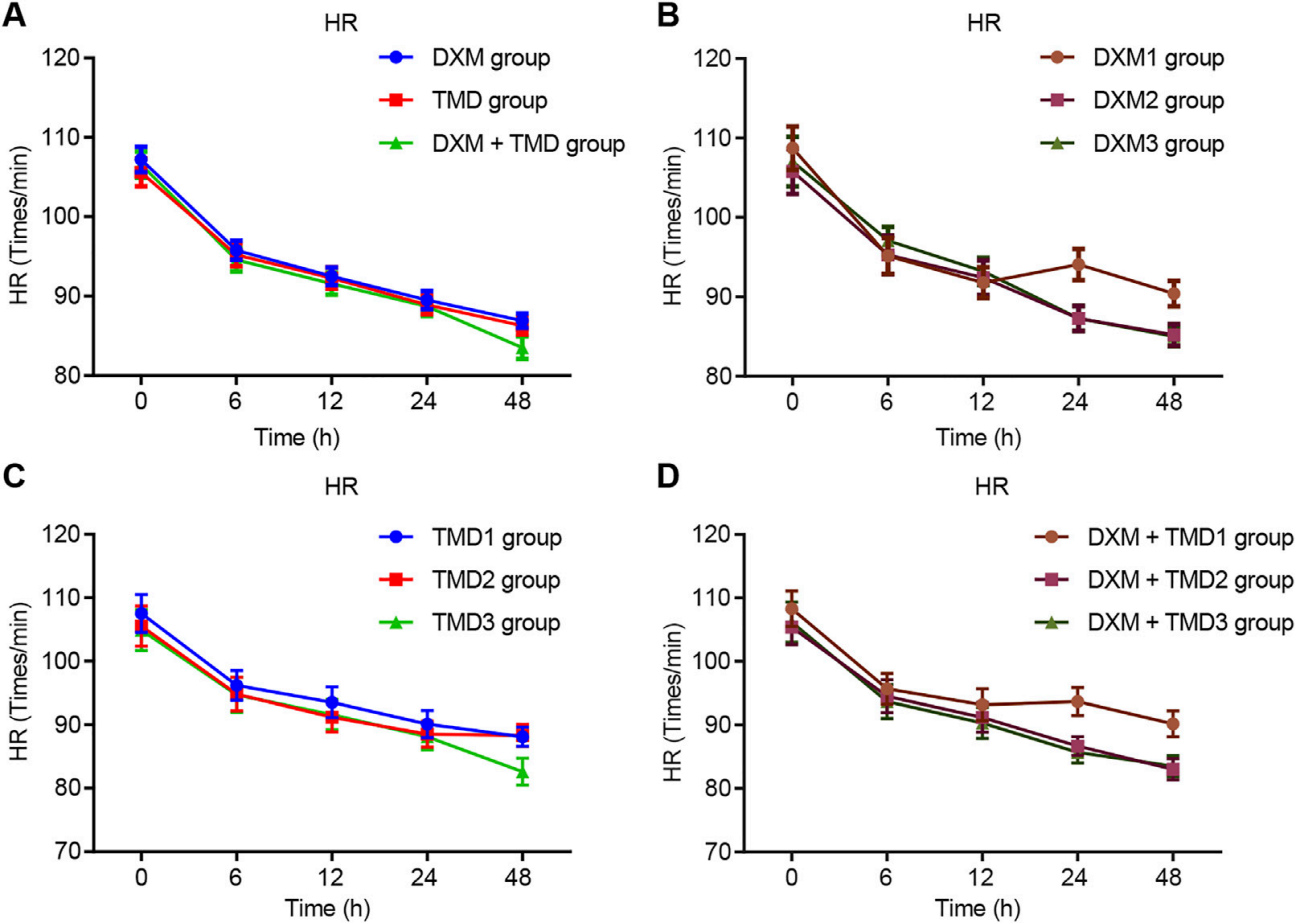
HR in three groups of patients with PIH.

**FIGURE 4:**
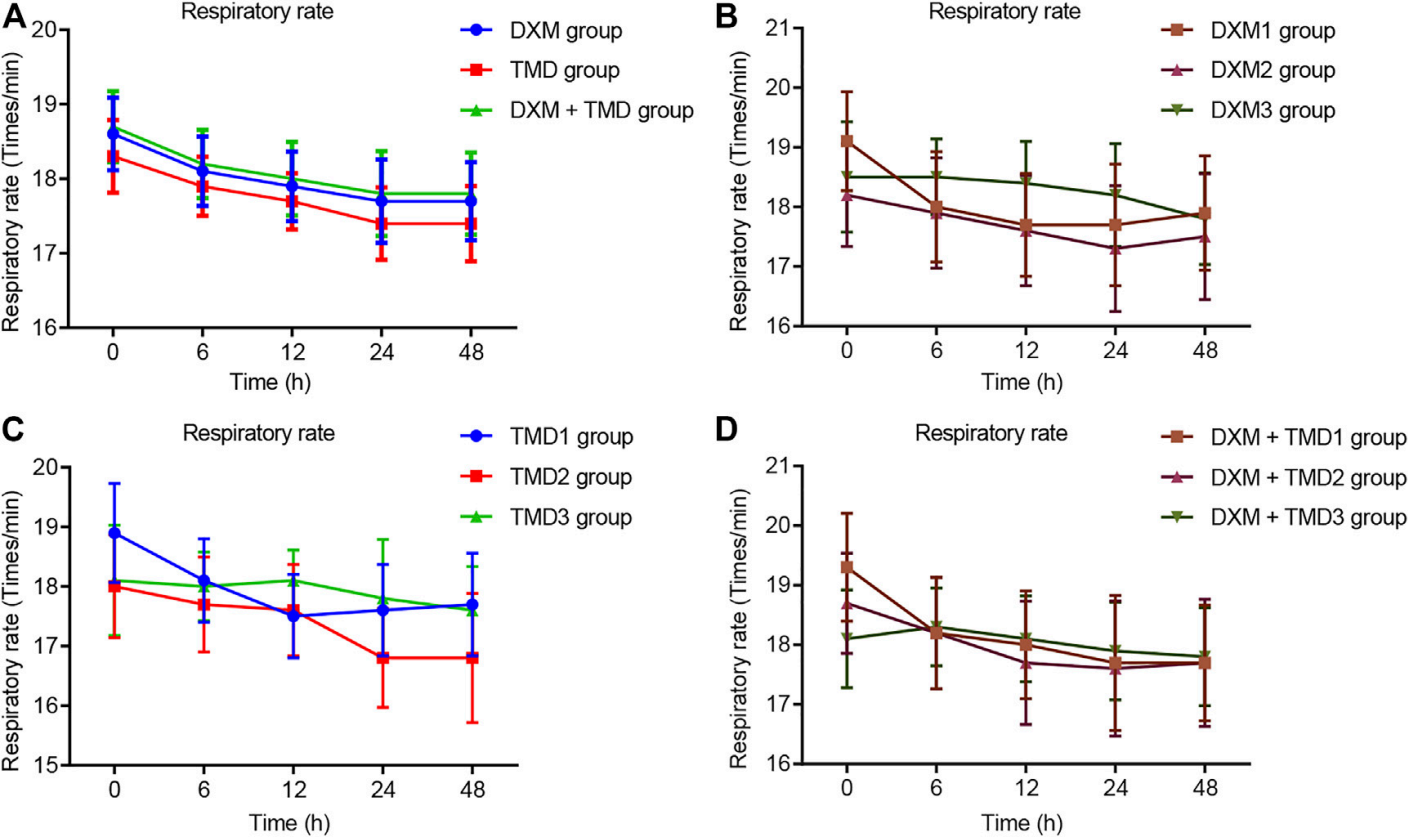
RR in three groups of patients with PIH.

**FIGURE 5:**
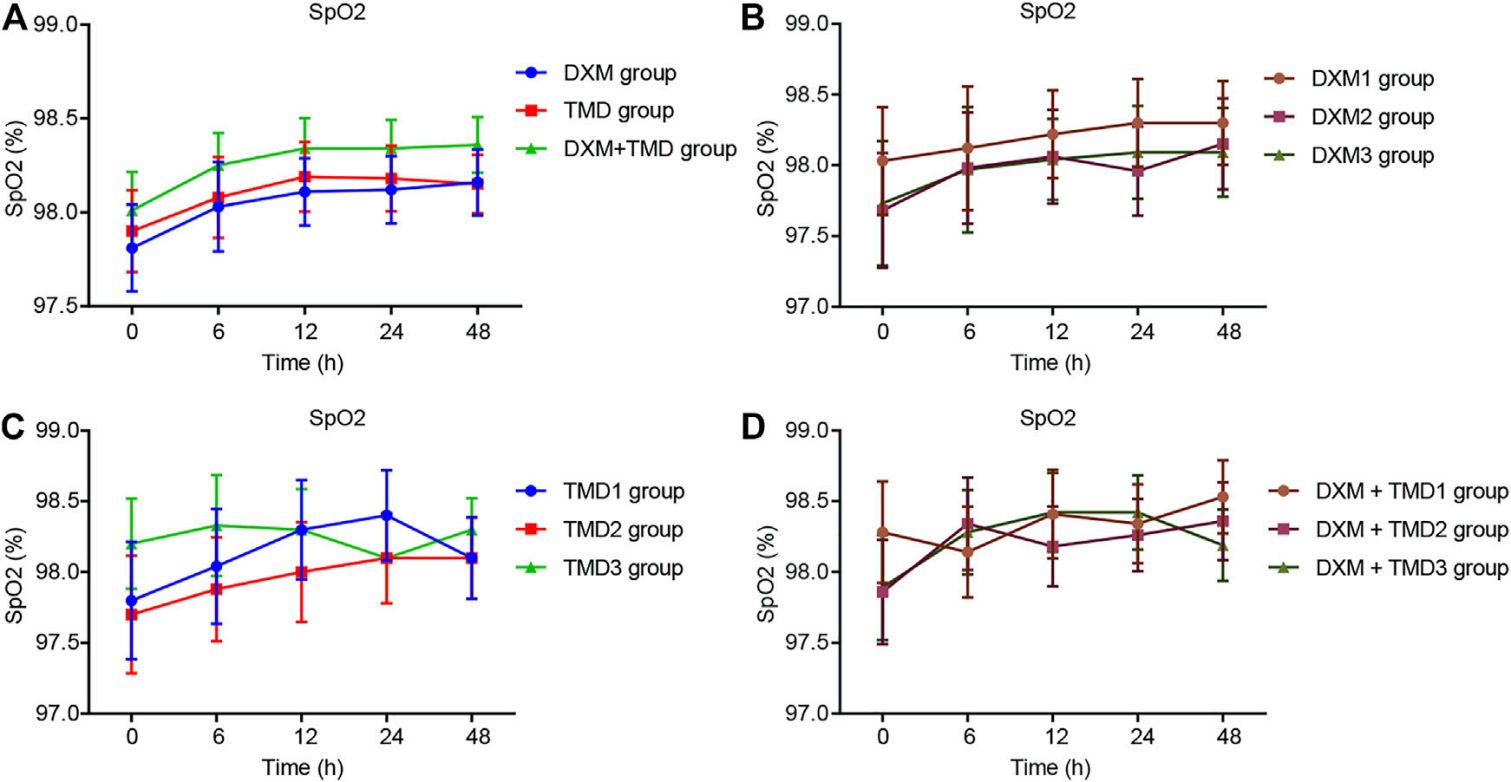
SpO_2_ in three groups of patients with PIH.

Compared with the MAP and HR of patients with PIH before anesthesia in the same group, the MAP and HR of patients with PIH in different groups at different time points showed varying degree of reduction (all *p* < 0.05). The MAP and HR of patients with PIH in the DXM + TMD group at 48 h-post surgery were lower than those of the DXM group (all *p* < 0.05), and there was no difference at other time points among other groups (all *p* > 0.05). RR and SpO_2_ were not statistically different at each time point of patients in the four groups (all *p* > 0.05)). At 24 and 48 h-post surgery, the MAP and HR of patients with PIH in the DXM group 1 were higher than those of the DXM group 2 and the DXM group 3 (all *p* < 0.05), there was no difference between the DXM group 2 and the DXM group 3 (*p* > 0.05). At the 48 h-post surgery, the MAP and HR of patients with PIH in the TMD group 1 and group 2 were higher than those in the TMD 3 group (all *p* < 0.05). At 24 and 48 h-post surgery, the MAP and HR of patients with PIH in the DXM + TMD group 1 were higher than those in the DXM + TMD group 2 and the DXM + TMD group 3 (all *p* < 0.05). There was no difference between the DXM + TMD group 2 and the DXM + TMD group 3 (*p* > 0.05). Comparisons of MAP, HR, RR and SpO_2_ at each time point of patients with PIH in DXM group 1, 2, 3, TMD group 1, 2, 3, and DXM + TMD group 1, 2, 3 are shown in Figures 2–5. These results showed that the MAP and HR were decreased by administration of DXM and TMD.

### Dexmedetomidine 1.0 µg/kg/h + Tramadol 700 mg and Dexmedetomidine 2.0 µg/kg/h + Tramadol 600 mg Show Stronger Sedative Effect in Patients With Pregnancy-Induced Hypertension

The pain and sedation scores of the patients with PIH at 24 h-post operation were evaluated and presented in Table 3. The results of the DXM groups showed that with the increased concentration of DXM or TMD, the Prince-Henry score was decreased, while the Ramsay score was increased. Compared with the DXM + TMD group 1, the Prince-Henry scores of the DXM + TMD groups 2 and 3 were lowered, while the Ramsay score was raised (all *p* < 0.05). There was no difference in the Prince-Henry and Ramsay scoring between the DXM + TMD groups 2 and 3 (all *p* > 0.05). These results revealed that the DXM + TMD group 2 (DXM 1.0 µg/kg/h + TMD 700 mg) and the DXM + TMD group 3 (DXM 2.0 µg/kg/h + TMD 600 mg) showed a stronger sedative effect compared with other treatments in patients with PIH.

**TABLE 3:** The pain and sedation scores of patients at 24 h after surgery in each group.

Group	Prince-Henry	Ramsay
DXM group	2.93 ± 0.86	2.38 ± 0.90
DXM sub-group 1	3.40 ± 0.78	2.05 ± 0.88
DXM sub-group 2	2.78 ± 0.70*	2.20 ± 0.72
DXM sub-group 3	2.60 ± 0.71*	2.88 ± 0.88*
TMD group	2.86 ± 0.81	2.44 ± 0.91
TMD sub-group 1	3.35 ± 0.77	2.13 ± 0.88
TMD sub-group 2	2.85 ± 0.70*	2.38 ± 0.93
TMD sub-group 3	2.38 ± 0.67*#	2.83 ± 0.78*
DXM + TMD group	1.81 ± 0.92	3.41 ± 1.23
DXM + TMD sub-group 1	2.31 ± 0.95*	2.82 ± 0.97*
DXM + TMD sub-group 2	1.69 ± 0.66*#&	3.64 ± 1.25*#&
DXM + TMD sub-group 3	1.39 ± 0.87*#&	3.76 ± 0.71*#&

Note: Subgroups of the DXM group and the TMD group: *n* = 40; the DXM + TMD sub-group 1 and the DXM + TMD sub-group 2: *n* = 39; the DXM + TMD sub-group 3: *n* = 38; the measurement data were expressed by mean ± standard deviation. Data of multiple groups were compared using one-way ANOVA; *, *p* < 0.05 compared with the DXM group 1; #, *p* < 0.05 compared with the TMD group 1; &, *p* < 0.05 compared with the DXM + TMD group 1; DXM, dexmedetomidine; TMD, tramadol.

### High Dexmedetomidine and Relatively Low Tramadol Lead to More Adverse Reaction in Patients With Pregnancy-Induced Hypertension

According to statistics, the results of adverse reaction of patients with PIH in each group showed that there was a slightly increased adverse reaction in the group 3 than the group 1 and 2 in the DXM group and the TMD group, but there was no significant difference (all *p* > 0.05). Compared with the DXM + TMD groups 1 and 2, the total adverse reaction rate was increased in the DXM + TMD group 3 (*p* < 0.05). There was no obvious difference in the incidence of total adverse reaction among the DXM + TMD groups 1 and 2, as well as the DXM group 1, 2, 3 or the TMD group 1, 2, 3 (all *p* > 0.05) (Table 4). These results indicated that patients with PIH in the DXM + TMD group 3 (DXM 2.0 µg/kg/h + TMD 600 mg) had higher adverse reaction than other groups.

**TABLE 4:** The adverse reaction of patients with PIH in each group.

Group	Nausea & vomitin g	Dizziness	Lethargy	Respiratory repression	Total adverse reaction rate [n (%)]
DXM group	17	13	10	4	44 (36.7%)
DXM sub-group 1	5	4	3	1	13 (32.5%)
DXM sub-group 2	4	4	3	1	12 (30.0%)
DXM sub-group 3	8	5	4	2	19 (47.5%)
TMD group	18	15	13	7	53 (44.2%)
TMD sub-group 1	5	5	3	1	14 (35.0%)
TMD sub-group 2	6	5	4	2	17 (42.5%)
TMD sub-group 3	4	5	6	4	22 (55.0%)
DXM + TMD group	20	17	13	6	56 (48.3%)
DXM + TMD sub-group 1	6	4	4	1	15 (38.5%)
DXM + TMD sub-group 2	5	5	3	1	14 (35.9%)
DXM + TMD sub-group 3	9	8	6	4	27 (71.1%)^*#&^

Note: Subgroups of the DXM group and the TMD group, *n* = 40; the DXM + TMD sub-group 1 and the DXM + TMD sub-group 2, *n* = 39; the DXM + TMD sub-group 3, *n* = 38; the data were expressed by ratio and percentage and analyzed by chi-square test; *p < 0.05 compared with the DXM group 1; #, *p* <0.05 compared with the TMD sub-group 1; &, *p* < 0.05 compared with the DXM + TMD sub-group 1. DXM, dexmedetomidine; TMD, tramadol; PIH, pregnancy-induced hypertension.

### Combination of Dexmedetomidine and Tramadol Increases IL-10, Decreases C-Reactive Protein, and Inhibits p-p38/MAPK

**FIGURE 6:**
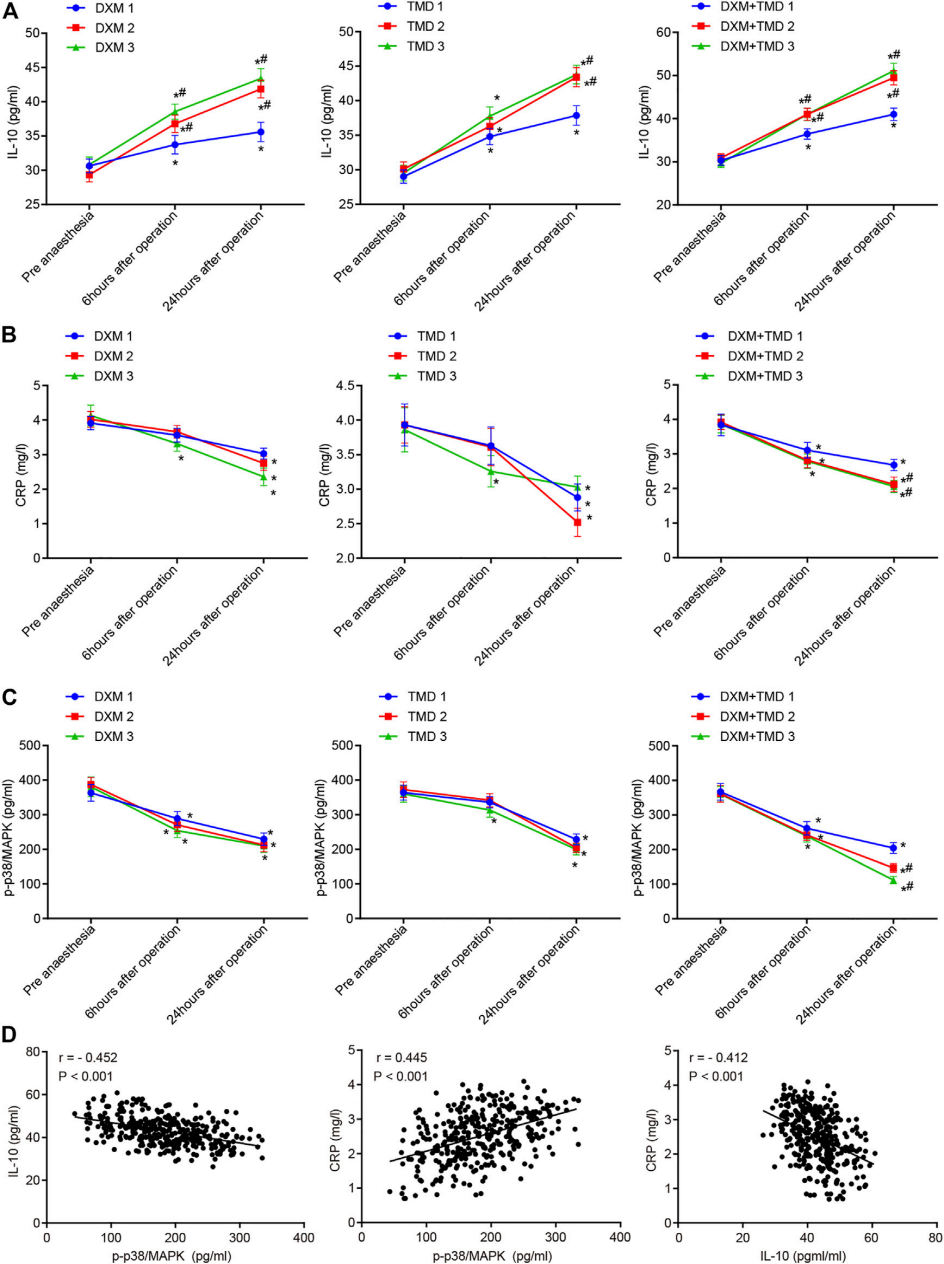
DXM and TMD increase IL-10 expression and inhibit p-p38/MAPK. **(A)** the levels of plasma IL-10 before anesthesia, 6 h and 24 h-post surgery in each group examined by ELISA; **(B)** the level of plasma CRP before anesthesia, 6 h and 24 h-post surgery in each group examined by ELISA; **(C)** the level of serum p-p38/MAPK before anesthesia, 6 h and 24 h-post operation in each group examined by ELISA; **(D)** the correlation among plasma IL-10, CRP and serum p-p38/MAPK at 24 h-post operation analyzed by Pearson correlation analysis. The measurement data were expressed as mean ± standard deviation, and data of multiple groups at different time points were compared using repeated measurement ANOVA. *, *p* < 0.05 compared with pre-anesthesia; #, *p* < 0.05 compared with subgroup 1 of each group; in each subgroup of DXM group and TMD group, *n* = 40; in the DXM + TMD groups 1 and 2, *n* = 39; in the DXM + TMD group 3, *n* = 38. CRP, C-reactive protein; p-p38/MAPK, p-p38/mitogen-activated protein kinase; IL-10, Interleukin 10; ELISA, enzyme linked immunosorbent assay; DXM, dexmedetomidine; TMD, tramadol.

Local inflammatory response, particularly the non-specific inflammatory factors IL-10 and CRP, played important roles in the etiology and pathogenesis of PIH. It has been reported that DXM could increase IL-10 expression (Li et al., 2016b; Li et al., 2015) and reduce CRP expression (Tong et al., 2017), thereby alleviating the degree of inflammation. The plasma IL-10 and CRP levels were measured by ELISA in this study (Figures 6A,B). Compared with pre-anesthesia, the plasma IL-10 was increased while plasma CRP was decreased at 24 h post surgery (all *p* < 0.05). The plasma IL-10 and CRP levels were compared at 24 h post surgery in the DXM + TMD groups 1, 2, and 3. Compared with the DXM + TMD group 1, the plasma IL-10 was increased but the plasma CRP was decreased in the DXM + TMD groups 2 and 3 (all *p* < 0.05). Meanwhile, DXM could reduce inflammatory factor levels by inhibiting p-p38/MAPK (Yamakita et al., 2017). To confirm this result, we measured serum p-p38/MAPK levels by ELISA (Figure 6C). As the concentration of DXM or TMD increased, serum p-p38/MAPK level was reduced. Compared with pre-anesthesia, serum p-p38/MAPK levels were lowered at 24 h after surgery in each group, and there was more obvious decrease in the DXM + TMD groups (all *p* <0.05). Correlation among plasma IL-10, CRP and serum p-p38/MAPK at 24 h after surgery was observed by Pearson correlation analysis. The results showed that serum p-p38/MAPK was negatively correlated with plasma IL-10 (*r* <= 0.452, *p* < 0.001) and positively correlated with plasma CRP (*r* = 0.350, *p* < 0.001), and plasma IL-10 was negatively correlated with CRP (*r* < −0.357, *p* < 0.001) (Figure 6D). These results showed that DXM could alleviate the inflammation by increasing the expression of IL-10 and decreasing CRP through inhibiting p-p38/MAPK.

## Discussion

PIH, a disease resulted from pregnancy, affects 12–15% of pregnant women (Kaur et al., 2012). However, there is no effective therapy so far (Han et al., 2018a). DXM has been reported to be able to relieve respiratory repression and has a stronger sedative and analgesic effect than other anesthetic drugs (Li et al., 2018). TMD is known to be effective in the treatment of various degrees of pain (Sadek et al., 2018). We hypothesized that combinational use of DXM and TMD would exert a beneficial effect for anesthesia on patients with PIH. The findings of our study showed that DXM combined with TMD in PCIA can low blood pressure, inhibit inflammation and promote sedation through inactivating the p-38-MMAPK signaling pathway and elevating IL-10.

We found that patients injected with DXM combined with TMD showed decreased blood pressure compared with patients used DXM or TMD alone in different groups at different time points. This result was in consistent with a previous meta-analysis which showed that patients treated with DXM had lower HR and MAP (Wang et al., 2018). Yue *et al.* has determined that DXM was a good controller for hypertension although it could cause an ephemeral increase of blood pressure (Yun et al., 2017). Our results also showed that high concentration of DXM might cause adverse reaction, however, appropriate amount of DXM was good for controlling hypertension. In addition, TMD administration has been demonstrated to lower systolic arterial blood pressure in rabbits (Egger et al., 2009).

A previous study demonstrated that decrease in blood pressure is an easily seen sympatholytic effect caused by DXM (Bedirli et al., 2017). Moreover, DXM has been demonstrated to improve the prognosis of patients suffered from PIH and displayed the role in controlling blood pressure as well as uterine bleeding in the process of cesarean section (Hariharan, 2017). Opioids are most frequently applied for PCIA, and TMD has been determined to have analgesic effects for pain after surgery (Schnabel et al., 2015). Besides, in patients who undergo cesarean section, TMD administration via PCIA provides efficient early postoperative analgesia (Chi et al., 2017), which was consistent with what we found that DXM combined with TMD in PCIA could decrease the blood pressure.

In addition, we found that patients injected with DXM 1.0 µg/kg/h + TMD 700 mg or DXM 2.0 µg/kg/h + TMD 600 mg showed increased level of IL-10 and decreased CRP in plasma by ELISA assay. IL-10 is a known anti-inflammatory factor that can prevent the occurrence and progression of atherosclerosis (Goldwater et al., 2019). It was reported that DXM could increase the expression of IL-10 (Li et al., 2015). Also, it has been confirmed that inflammation can be decreased and relieved by DXM in rats (Tan et al., 2018). CRP is an inflammatory biomarker (Thiele et al., 2015). The expression of CRP was reduced by DXM administration (Han et al., 2018b). TMD had the function of suppressing inflammatory factors, like tumor necrosis factor alpha (TNF-α) released in monocyte cultures (Lamana et al., 2017). Furthermore, TMD combined with ibuprofen was previously revealed to increase analgesia and inhibit inflammation, which has been determined in experiment based on animals with pains and inflammatory symptoms (Suthakaran et al., 2017). Therefore, the results demonstrated that DXM combined with TMD could increase the expression of IL-10 and reduce CRP expression, thus suppressing the inflammatory responses, that could help alleviating PIH.

Moreover, the study showed that the combination of DXM with TMD might exert anti-inflammatory and sedative effects in patients *via* inhibiting the p38-MAPK signaling pathway and increasing the expression of IL-10. It has been reported that DXM can lower the levels of inflammatory factor by suppressing p-p38/MAPK (Yamakita et al., 2017), which is consistent with our findings. Potthoff SA *et al.* has suggested that p38-MAPK is an important regulator for blood pressure as well as vascular injury and could be a pharmaceutical target (Potthoff et al., 2016). DXM was demonstrated to be able to alleviate systemic inflammatory response syndrome and fever (Tan et al., 2018), suggesting that DXM could effectively inhibit inflammation. IL-10 is widely known as an anti-inflammatory factor (Couper et al., 2008). Therefore, it is the anti-inflammatory response of DXM might be mediated by the interaction with IL-10. Moreover, DXM was reported to attenuate inflammation by decreasing the expression of IL-6, a proinflammation factor (Zeng et al., 2016). A previous study showed that TMD exerted important effect on pain relief (Sayed et al., 2018). It has been demonstrated that TMD might alleviate disease through decreasing activity of mTOR signaling pathways (Pourtalebi Jahromi et al., 2018). Thus, our finding suggests that DXM with TMD could exert their anti-inflammatory and sedative effects *via* inhibiting the p38-MAPK signaling pathway. However, due to the limited experimental conditions, clinical analysis will be conducted in the future to verify our results and find out the values of DXM in combination with TMD in the treatment of PIH. Moreover, more efforts are needed to detect the dose effects of DXM and TMD and evaluate the anesthetic effect of DXM and TMD is additive or synergistic .

In conclusion, the findings in this present study demonstrated that low concentration of DXM in combination with TMD in PCIA improves the postoperative sedation and analgesia in patients with PIH. The combination of DXM and TMD could effectively decrease blood pressor, inhibit inflammation by inhibition of p38/MAPK signaling pathway and upregulation of IL-10. This study provided an option for postoperative care for PIH.

## Data Availability

The raw data supporting the conclusions of this article will be made available by writing to the corresponding author, without undue reservation.
